# External Validation of aMAP Hepatocellular Carcinoma Risk Score in Patients With Chronic Hepatitis B-Related Cirrhosis Receiving ETV or TDF Therapy

**DOI:** 10.3389/fmed.2021.677920

**Published:** 2021-08-04

**Authors:** Honglian Gui, Yan Huang, Gangde Zhao, Lichang Chen, Wei Cai, Hui Wang, Qing Guo, Qing Xie

**Affiliations:** Department of Infectious Diseases, Ruijin Hospital, Shanghai Jiao Tong University School of Medicine, Shanghai, China

**Keywords:** hepatocellular carcinoma, liver cirrhosis, risk score, hepatitis B virus, diabetes mellitus, nucleos(t)ide analogs

## Abstract

**Background and Aim:** A prediction model of hepatocellular carcinoma (HCC) risk in patients with chronic liver diseases, based on age, male sex, albumin-bilirubin, and platelets (aMAP), has been previously reported. We validated the aMAP score and compared its performance to those of other risk scores in an independent at-risk cohort.

**Methods:** Treatment-naïve patients with chronic hepatitis B-related compensated cirrhosis who received entecavir or tenofovir monotherapy for at least 12 months were enrolled in this study. The performances of the aMAP and other HCC risk scores were assessed using Harrell's c-index, and predefined cut-off values were evaluated using survival analysis.

**Results:** Of the 1,042 patients, 131 (12.6%) developed HCC during a median follow-up of 41 months. The aMAP score provided the highest Harrell's c-index (0.724), followed by CAMD (0.719), mPAGE-B (0.719), and PAGE-B (0.695). The 5-year cumulative HCC probabilities were 2.9% for patients with a low aMAP score (<50), 11.2% for patients with an intermediate aMAP score (50–60), and 27.9% for patients with a high aMAP score (>60). Using both aMAP and mPAGE-B, 11.6% of patients were identified as low risk with a negative predictive value of 98.2% for not developing HCC within 5 years. Patients with aMAP >60 and diabetes exhibited an extremely high risk of HCC, with a cumulative incidence of 49.3% at 5 years. The predictive performance of aMAP with a reassessment at 1 year after the initiation of antiviral therapy outperformed the predictive performance of aMAP at enrollment.

**Conclusions:** The aMAP score accurately predicted the risk of HCC in at-risk patients with compensated cirrhosis undergoing antiviral therapy. A combination of the aMAP score and diabetes status could further stratify the risk of HCC.

## Introduction

Chronic hepatitis B virus (HBV) infection represents a serious public health problem and is one of the major causes of hepatocellular carcinoma (HCC) worldwide. Among patients with chronic hepatitis B (CHB), the risk of HCC development increases with the severity of cirrhosis (1). The annual HCC incidence for patients with CHB without cirrhosis is ~0.01–1.4%, while the HCC incidence increases to 0.9–10.8% in patients with cirrhosis (2, 3). With the introduction of nucleos(t)ide analog (NA) therapy over the past two decades, long-term viral suppression has emerged as the most dominant modifier of HCC in patients with CHB (4). In patients with cirrhosis, NA therapy with entecavir (ETV) or tenofovir disoproxil fumarate (TDF) results in a significant reduction (~30%) in HCC risk (3). However, HCC risk cannot be completely eliminated in patients receiving NA therapy, especially in patients with cirrhosis (5, 6). The high risk of HCC highlights the need for disease management in this at-risk population.

Early detection of HCC by periodic surveillance is critical for improving patient outcomes. HCC surveillance is currently recommended in all patients with cirrhosis; however, the risk of HCC varies widely among patients. Thus, an individual HCC risk prediction is of importance for implementing feasible and effective HCC screening. Therefore, numerous HCC risk scores have been developed. Early scores are developed in untreated patients with CHB and include factors such as age, sex, HBV DNA, core promoter mutations, and the cirrhosis-HCC score (GAG-HCC) (7), the Chinese University of Hong Kong-HCC score (CU-HCC) (8), risk estimation for HCC in CHB score (REACH-B) (9), liver stiffness measurement-HCC score (LSM-HCC) (10), and real-world score for HCC (RWS-HCC) (11). However, such scores may have led to an overestimation of HCC incidence in patients undergoing NA therapy. Therefore, several scores have been developed in patients with CHB undergoing NA therapy, including the modified REACH-B score (mREACH-B) (12), platelets, age, and gender score (PAGE-B) (13), cirrhosis, age, male sex, and diabetes mellitus score (CAMD) (14), and the modified PAGE-B score (mPAGE-B) (15). Recently, a novel scoring system that includes age, male sex, albumin-bilirubin, and platelets (aMAP) has been proposed, and its performance in assessing the 5-year HCC risk has been validated in several cohorts with different etiologies and ethnicities (16), but its performance for late HCC after a longer period of treatment warrants further research.

As various HCC risk scores are available and patients with CHB are a heterogeneous group, it is not easy for clinicians to determine if these scores can be applied in their clinical practice effectively and which HCC risk score should be used for specific patients. Moreover, the potential clinical utility of these HCC risk scores in patients with cirrhosis remains unclear, as patients with cirrhosis are excluded from many studies, and there has been no study focusing on this at-risk population (17). In this study, we externally validated the predictability of the aMAP score using an independent cohort of treatment-naïve patients with CHB-related compensated cirrhosis who were receiving ETV or TDF at different follow-up timepoints, especially outside its original intended time horizon. We compared the performance of the aMAP score with that of other risk scores. Furthermore, we incorporated other emerging risk factors to the aMAP score to enhance its predictive ability and clinical relevance. Moreover, we explored whether its predictability would change with re-assessment of the score during therapy.

## Materials and Methods

The study protocol was conducted in accordance with the ethical guidelines of the Declaration of Helsinki 1975 and was approved by the Research Ethics Committee of Ruijin Hospital (No.2019-202). As this was a retrospective analysis of de-identified medical records, patient informed consent was not obtained.

### Study Design

Patients with CHB-related compensated cirrhosis who started antiviral therapy with ETV or TDF at Ruijin Hospital from January 2005 to December 2018 were consecutively screened for eligibility for this study. The inclusion criteria were as follows: (i) HBsAg positive for at least 6 months; (ii) ≥18 years old at therapy initiation; (iii) treatment-naïve patients who started ETV 0.5 mg/d or TDF 300 mg/d as the first-line antiviral regiment; (iv) patients with compensated cirrhosis; and (v) available clinical data to calculate the aMAP, PAGE-B, mPAGE-B, and CAMD scores at baseline and 1-year after antiviral therapy. The exclusion criteria were as follows: (i) follow-up duration of <12 months; (ii) history of decompensated cirrhosis, HCC, liver transplantation, or stem cell transplantation at enrollment; (iii) co-infection with other viral hepatitis or human immunodeficiency virus; (iv) hepatic decompensation, HCC, or death within 12 months of enrollment; and (v) continuous interferon therapy for at least 4 weeks at any time. Patients with decompensated cirrhosis were excluded due to the short survival associated with that condition.

### Data Collection and Follow-Up

All data (demographic, biochemical, virological, histological, and radiological features) were extracted retrospectively from the hospital electronic patient database. Baseline data were defined as those at the time of ETV or TDF initiation. Baseline clinical and laboratory parameters, including age; sex; diagnosis of diabetes mellitus (DM); levels of alanine aminotransferase (ALT), aspartate aminotransferase (AST), albumin, total bilirubin, hepatitis B e antigen, and HBV DNA; and platelet count were collected. All patients were regularly followed up every 3–6 months. The primary outcome was HCC development. Patients who underwent liver transplantation during the study period were also recorded. The follow-up endpoint was the date of HCC diagnosis, liver transplantation, or the last outpatient clinic visit in the absence of HCC development. Patients lost to follow-up were censored to the last documented visit. A total of 253 (24.2%) patients were lost to follow-up at the end of the study.

### Diagnoses and Clinical Evaluations

The presence of cirrhosis was defined as any one of the following: (i) liver biopsy showing cirrhosis (Ishak score ≥5 or Metavir score = 4); (ii) liver stiffness measurement (by fibroscan) ≥12.0 kPa when ALT ≤ 40 U/L and the total bilirubin was normal, or ≥17.0 kPa when ALT <200 U/L and the total bilirubin was normal (18); (iii) AST-to-platelet ratio index (APRI) ≥2.0; (iv) fibrosis-4 (FIB-4) ≥3.25; and/or (v) abdominal imaging (modalities with coarse liver echotexture or nodular, parenchymal, or morphological abnormalities and signs of gastroesophageal varices).

The diagnosis of decompensated cirrhosis was based on the presence of pre-existing ascites, upper gastrointestinal (esophageal and/or gastroduodenal) bleeding, spontaneous bacterial peritonitis, hepatorenal syndrome, and hepatic encephalopathy.

HCC was diagnosed based on histological evidence or typical radiological features, as follows: (i) dynamic computed tomography (CT) and/or magnetic resonance imaging (MRI) findings (nodule >1 cm with arterial hypervascularity and portal/delayed-phase washout); and/or (ii) tumor staining by lipiodol on hepatic angiography.

The presence of DM was diagnosed based on medical record of any anti-diabetic agents, and/or hemoglobin A_1c_ ≥6.5%, and/or fasting plasma glucose ≥7 mmol/L or 2-h plasma glucose ≥11.1 mmol/L during an oral glucose tolerance test (19).

### HCC Risk Scores and Cut-Off Points for Risk Stratification

Four published HCC risk scores (PAGE-B, mPAGE-B, CAMD, and aMAP) based on the clinical characteristics and laboratory parameters were used in this study. Patients were then categorized into low-, intermediate-, and high-risk HCC stratification groups according to the cut-off points as previously described as shown in Supplementary Table 1 (13–16). It should be noted that most cut-off points derived from 5-year HCC incidences might not be the most discriminatory at other follow-up time points.

In addition to the baseline and on-treatment (1 year) scores, the on-treatment change in aMAP score, defined as the aMAP score at 1 year minus the aMAP score at baseline, was calculated and presented as ΔaMAP.

### Statistical Analysis

Statistical analyses were performed using SPSS software (version 23 for Windows; IBM Corp., Armonk, NY) and R software (version V.3.6.2, http://cran.r-project.org). Two-sided *P*-values ≤ 0.05 were statistically significant.

Continuous variables were presented as mean ± standard deviation (SD) or median (interquartile range, IQR) and were compared using unpaired *t*-tests or Mann-Whitney tests, as appropriate. Frequency variables were expressed as numbers and percentages and were compared using the chi-squared test or Fisher's exact test, as appropriate. Univariable and multivariable cox proportional hazards regression models were used to estimate the effect of various variables on the hazard of HCC occurrence. Variables with *P* < 0.1 on univariate analysis were entered into multivariate analysis. Hazard ratios (HR) and their 95% confidence intervals (CIs) along with corresponding *P*-values were presented. The cumulative probabilities of HCC occurrence at different timepoints (3, 5, 7, and 10 years, respectively) were estimated by the Kaplan-Meier method and compared with the log-rank test. The predictive performances of the aMAP score and other scores were assessed by calculating Harrell's c-index. Statistical differences in Harrell's c-indices between aMAP and the other scores were evaluated using the bootstrap method with 1,000 re-samplings. A 95% CI not containing zero was regarded as indicative of a significant difference in Harrell's c-index between the two models. The *p*-value was calculated by using the Z testing method. To assess the diagnostic accuracy of the suggested cut-off points, the cut-off of low risk was used to report negative predictive value (NPV) with 95% CI, while the cut-off of high risk was used to define the positive predictive value (PPV) with 95% CI.

## Results

### Baseline Clinical Characteristics

**Figure 1:**
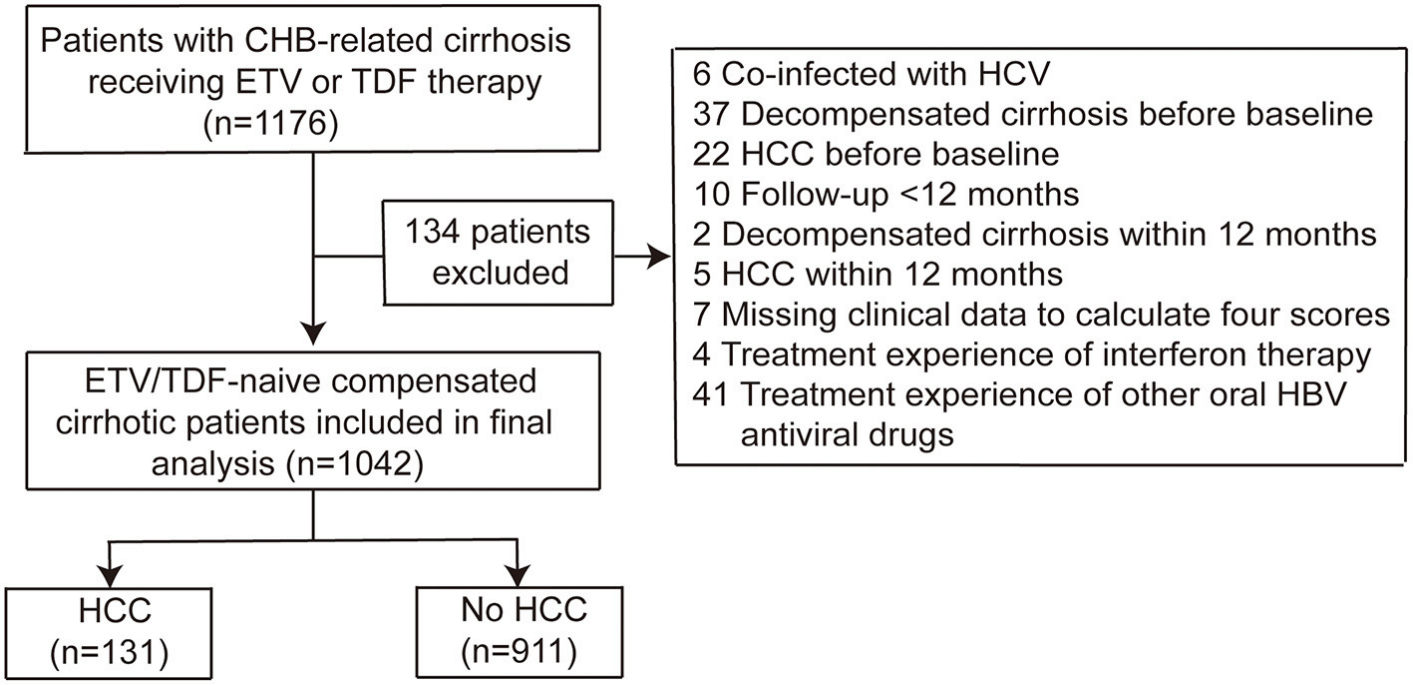
Flow chart of patients with chronic hepatitis B-related cirrhosis receiving ETV or TDF therapy.

We identified 1,176 patients with CHB-related cirrhosis who received ETV or TDF treatment; 134 patients were excluded according to the exclusion criteria, the majority due to pre-existing decompensation and HCC before baseline, and having treatment experience of other nucleotide antiviral drugs (Figure 1). Finally, a total of 1,042 NA-naïve patients with compensated cirrhosis were included and analyzed. The baseline characteristics are presented in Table 1. The mean age of the patients was 48.4 ± 11.7 years, and 67.3% (701/1,042) of the patients were male. Overall, 85 (8.2%) patients had diabetes. ETV and TDF were initiated as a first-line antiviral regimen in 937 (89.9%) and 105 (10.1%) patients, respectively. All patients had cirrhosis at enrollment, including 191 (18.3%) patients whose diagnosis was based on a liver biopsy. The mean aMAP, CAMD, PAGE-B, and mPAGE-B scores were 57.8, 14.0, 15.7, and 11.8, respectively.

**Table 1:** Baseline and on-treatment characteristics of the study population.

**Variables**	**Overall (*N* = 1,042)**	**Patients without HCC (*N* = 911)**	**Patients with HCC (*n* = 131)**	***P*-value**
Age, y	48.4 ± 11.7	47.4 ± 11.7	55.3 ± 8.39	<0.001
Male gender, *n* (%)	701 (67.3)	604 (66.3)	97 (74.0)	0.096
Diabetes mellitus, *n* (%)	85 (8.2)	62 (6.81)	23 (17.6)	<0.001
HBeAg-positive, *n* (%)	439 (42.1)	380 (41.7)	59 (45.0)	0.476
HBV DNA, log_10_IU/ml	5.11 ± 1.53	5.12 ± 1.56	5.07 ± 1.28	0.737
**AVT type**				0.003
ETV, *n* (%)	937 (89.9)	810 (88.9)	127 (96.9)	
TDF, *n* (%)	105 (10.1)	101 (11.1)	4 (3.1)	
ALT, IU/mL	83.2 ± 125	86.1 ± 131	63.2 ± 65.7	0.002
AST, IU/mL	67.7 ± 85.5	68.5 ± 88.0	61.7 ± 65.7	0.287
Platelet count, ×10^9^/L	113.0 ± 48.9	115.0 ± 48.3	98.0 ± 50.6	<0.001
Albumin, g/L	40.2 ± 5.58	40.4 ± 5.58	38.7 ± 5.31	<0.001
Total bilirubin, μmol/L	24.4 ± 27.1	23.7 ± 25.3	29.3 ± 36.8	0.094
aMAP score	57.8 ± 7.16	57.1 ± 7.04	62.6 ± 6.06	<0.001
CAMD score	14.0 ± 2.33	13.7 ± 2.27	15.6 ± 1.98	<0.001
PAGE-B score	15.7 ± 4.14	15.4 ± 4.11	18.1 ± 3.54	<0.001
mPAGE-B score	11.8 ± 3.15	11.5 ± 3.13	13.8 ± 2.47	<0.001
1-year aMAP score	56.7 ± 7.25	55.9 ± 7.11	61.9 ± 5.98	<0.001
ΔaMAP ≥ 0, *n* (%)	407 (39.1)	348 (38.2)	59 (45.0)	0.151

*Categorical variables were presented as frequency (percentage). Follow-up duration was expressed in median (interquartile range). Other continuous variables were expressed in mean ± standard deviation. ΔaMAP presented the on-treatment change in aMAP score (from 1 year of treatment to baseline)*.

*ALT, alanine aminotransferase; AST, aspartate aminotransferase; AVT, antiviral treatment; CI, confidence interval; ETV, entecavir; HBeAg, hepatitis B e antigen; TDF, tenofovir*.

### Clinical Events During Follow-Up

The median follow-up until HCC, liver transplantation or censoring of the 1,042 patients was 41 months (IQR: 27–64 months), during which 131 (12.6%) patients developed HCC, and none of these patients underwent liver transplantation. The cumulative incidence (95% CI) of HCC development at 3, 5, 7, and 10 years was 7.7% (5.9–9.6%), 16.6% (13.5–19.7%), 23.1% (18.5–27.3%), and 28.0% (21.7–33.9%), respectively. The baseline characteristics of patients who developed HCC and those who did not are compared in Table 1. Patients with HCC were older, had a higher rate of DM, and had lower ALT, albumin, and platelet counts than patients without HCC (all *P* < 0.05).

### Predictors of HCC Development

As shown in Table 2, univariate analysis revealed that male sex, age, the presence of DM, serum albumin, ALT, total bilirubin, and platelet count were significantly associated with HCC development (all *P* < 0.05). In multivariate analysis, male sex (HR: 1.888; 95% CI: 1.267–2.814), age (HR: 1.054; 95% CI: 1.037–1.072), DM (HR: 2.235; 95% CI: 1.416–3.529), ALT (HR: 0.996; 95% CI: 0.991–1.000), total bilirubin (HR: 2.235; 95% CI: 1.416–3.529), and platelet count (HR: 0.995; 95% CI: 0.991–1.000) were the independent predictors for HCC development.

**Table 2:** Predictors of HCC development.

	**Univariate**		**Multivariate**	
**Variables**	**HR (95% CI)**	***P*-value**	**HR (95% CI)**	***P*-value**
Gender (male vs. female)	1.59 (1.08–2.36)	0.016	1.888 (1.267–2.814)	0.002
Age (per year increase)	1.06 (1.04–1.08)	<0.001	1.054(1.037–1.072)	<0.001
Diabetes (yes vs. no)	3.03 (1.93–4.76)	<0.001	2.235(1.416–3.529)	<0.001
Platelet count (per 10^9^/L)	0.992 (0.988–0.996)	<0.001	0.995(0.991–1.000)	0.039
ALT (per IU/L)	0.998 (0.995–1.00)	0.048	0.996(0.991–1.000)	0.030
AST (per IU/l)	1.00 (0.997–1.00)	0.805	-	-
Albumin (per g/L)	0.943 (0.914–0.974)	<0.001	0.998(0.961–1.036)	0.894
Total bilirubin (per μmol/L)	1.01 (1.00–1.01)	0.007	1.011(1.004–1.018)	0.002
HBeAg status (positive vs. negative)	1.03 (0.731–1.46)	0.861	-	-
HBV DNA (per log_10_IU/ml)	0.971 (0.868–1.09)	0.606	-	-

*ALT, alanine aminotransferase; AST, aspartate aminotransferase; CI, confidence interval; HBeAg, hepatitis B e antigen*.

### Performance of aMAP Score at Baseline

The aMAP score provided the highest Harrell's c-index to predict the development of HCC (0.724; 95% CI: 0.701–0.747), followed by the CAMD (0.719; 95% CI: 0.696–0.741), mPAGE-B (0.719; 95% CI: 0.696–0.741), and PAGE-B (0.695; 95% CI: 0.671–0.720) scores (Table 3). The aMAP score showed the similar performance to the CAMD and mPAGE-B scores and the better performance than the PAEG-B score.

**Table 3:** Comparison of the predictive performances of HCC risk scores at baseline.

**HCC risk score**	**Harrell's c-index**	**95% CI**
aMAP	0.724	0.701–0.747
CAMD	0.719	0.696–0.741
PAGE-B	0.695	0.671–0.720
mPAGE-B	0.719	0.696–0.741
**Comparison**	**Difference between Harrell's c-indices of models**	**95% CI^a^**
aMAP vs. CAMD	−0.005	−0.043–0.034^a^
aMAP vs. PAGE-B	−0.029	−0.055– −0.003
aMAP vs. mPAGE-B	−0.006	−0.021–0.010^a^

*HCC, hepatocellular carcinoma; CI, confidence interval*.

a *If 95% CI interval contains zero, there is no significant difference between two models*.

### Risk Stratification According the Pre-defined Cut-Off Points

**Figure 2:**
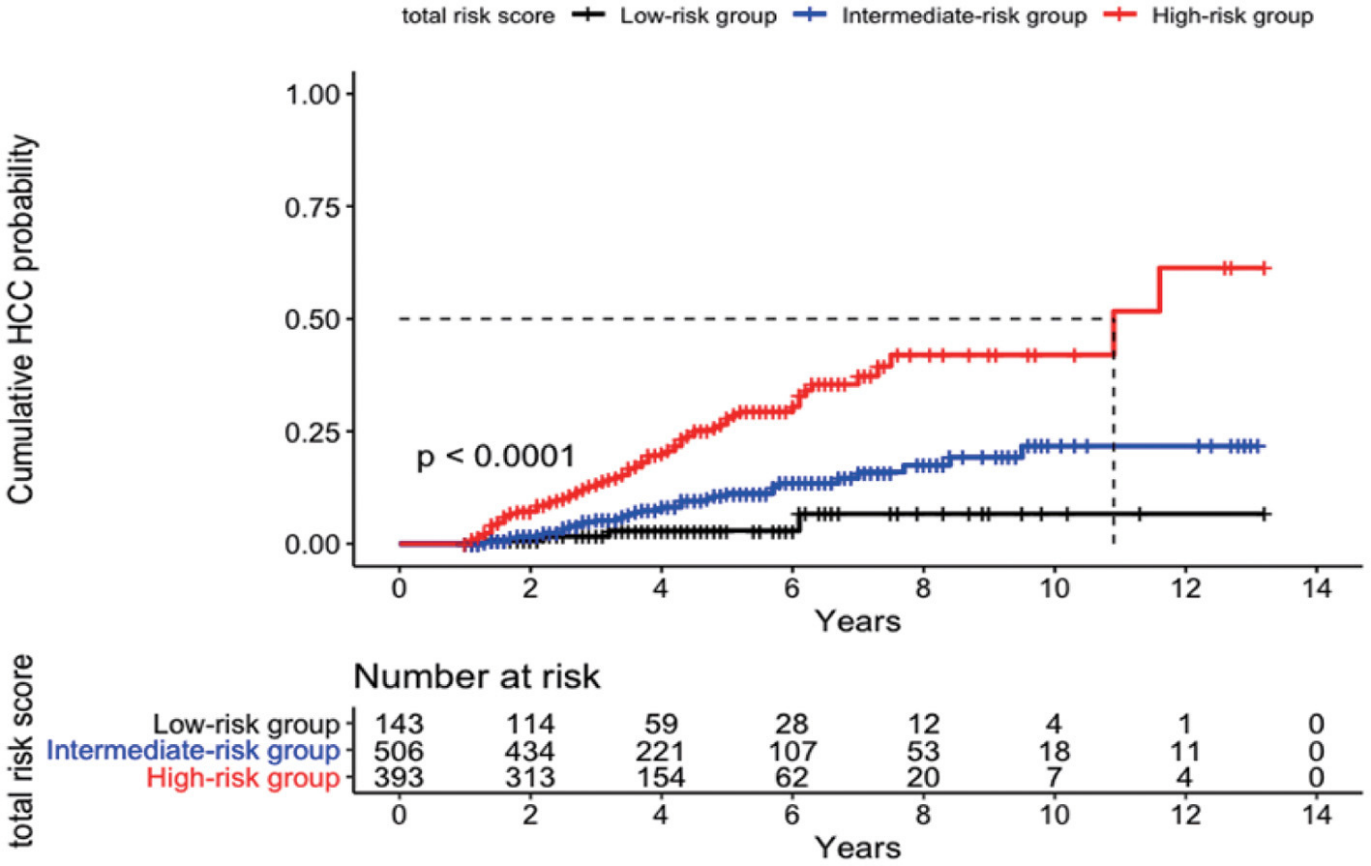
Cumulative probability of development of HCC according to risk stratification by the aMAP score.

The cumulative incidences (95% CI) of HCC development at 3, 5, 7, and 10 years were 1.7% (0.0–3.9%), 2.9% (0.0–6.2%), 6.7% (0.0–14.2%), and 6.7% (0.0–14.2%) in the low-risk group (aMAP score <50; *n* = 143), 5.2% (3.0–7.3%), 11.2% (7.4–14.9%), 15.9% (10.3–21.2%), and 21.7% (13.0–29.6%) in the intermediate-risk group (aMAP score 50–60; *n* = 506), and 13.1% (9.4–16.7%), 27.9% (21.8–33.6%), 37.3% (28.6–45.0%), and 42.0% (31.3–51.0%) in the high-risk group (aMAP score >60; *n* = 393), respectively (*p* = 0.04 between the low- and intermediate-risk groups; *p* < 0.0001 between the intermediate- and high-risk groups) (Figure 2).

### Diagnostic Accuracy in the Low-Risk Group by aMAP Score

The aMAP low-risk group achieved NPVs for the development of HCC within 3 and 5 years of 98.3% (95% CI: 96.0–100.0%) and 96.6% (95% CI: 92.8–100.0%), respectively. NPVs of the low-risk groups classified by other HCC risk scores alone or defined using both the “AND” and “OR” approaches by aMAP and other scores are described in Table 4. When patients were stratified using the aMAP and mPAGE-B scores, 121 (11.6%) patients were classified as low risk with NPVs for the development of HCC within 3 and 5 years of 100% (95% CI: 100.0–100.0%) and 98.2% (95% CI: 94.7–100.0%), respectively.

**Table 4:** Diagnostic accuracy on HCC development in 3 and 5 years by low- and high-risk cut-off of aMAP and other risk scores.

**Risk score**	**Low-risk groups**			**High-risk groups**		
	**Pts, *n* (%)**	**NPV (%) (95% CI)**		**Pts, *n* (%)**	**PPV (%) (95% CI)**	
		**3 years**	**5 years**		**3 years**	**5 years**
aMAP alone	143 (13.7)	98.3 (96.0–100.0)	96.6 (92.8–100.0)	393 (37.7)	12.7 (9.0–16.3)	25.7 (19.8–31.6)
PAGE-B alone	66 (6.3)	98.0 (94.1–100.0)	95.1 (88.2–100.0)	368 (35.3)	13.0 (9.2–16.8)	26.6 (20.5–32.6)
mPAGE-B alone	154 (14.8)	99.3 (97.9–100.0)	97.8 (94.6–100.0)	431 (41.4)	13.1 (9.5–16.8)	24.9 (19.4–30.4)
CAMD alone	0 (0)	-	-	510 (48.9)	11.9 (8.7–15.1)	24.8 (19.8–29.9)
aMAP and PAGE-B	61 (5.9)	97.8 (93.6–100.0)	94.8 (87.6–100.0)	294 (28.2)	12.6 (8.4–16.7)	26.6 (19.8–33.3)
aMAP or PAGE-B	148 (14.2)	98.4 (96.1–100.0)	96.7 (92.9–100.0)	467 (44.8)	13.0 (9.6–16.4)	25.9 (20.5–31.2)
aMAP and mPAGE-B	121 (11.6)	100.0 (100.0–100.0)	98.2 (94.7–100.0)	350 (33.6)	14.1 (10.0–18.2)	27.4 (21.0–33.8)
aMAP or mPAGE-B	154 (14.8)	99.3 (97.9–100.0)	97.8 (94.6–100.0)	474 (45.5)	12.0 (8.8–15.3)	23.8 (18.6–28.9)
aMAP and CAMD	0 (0)	-	-	317 (30.4)	13.5 (9.3–17.6)	28.1 (21.3–34.9)
aMAP or CAMD	143 (13.7)	98.3 (96.0–100.0)	96.6 (92.8–100.0)	586 (56.2)	11.6 (8.7–14.5)	23.6 (19.0–28.2)

*CI, confidence interval; NPV, negative predictive value; PPV, positive predictive value*.

*Low-risk groups include patients with score below the reported low-risk cut-off; high-risk groups include patients with score above the reported high-risk cut-off*.

### Diagnostic Accuracy in the High-Risk Group by aMAP Score

The aMAP high-risk group achieved PPVs of developing HCC within 3 or 5 years of 12.7% (95% CI: 9.0–16.3%) and 25.7% (95% CI: 19.8–31.6%), respectively. PPVs of the high-risk groups classified by other HCC risk scores alone or defined using both the “AND” and “OR” approaches by aMAP and other scores are listed in Table 4. When patients were stratified using the aMAP and mPAGE-B scores, 350 (33.6%) patients were classified as high risk with PPVs for the development of HCC within 3 and 5 years of 14.1% (95% CI: 10.0–18.2%) and 27.4% (95% CI: 21.0–33.8%), respectively.

### Cumulative Incidence of HCC Stratified by aMAP and DM

**Figure 3:**
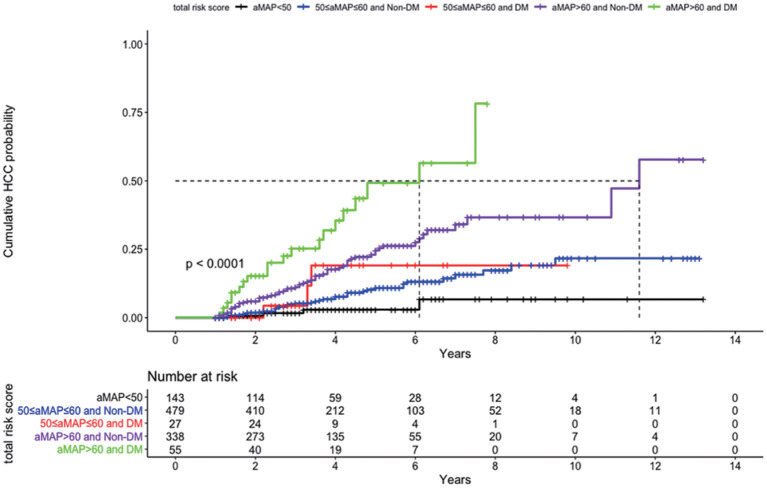
Cumulative probability of development of HCC according to risk stratification by combination of aMAP and DM.

**Figure 4:**
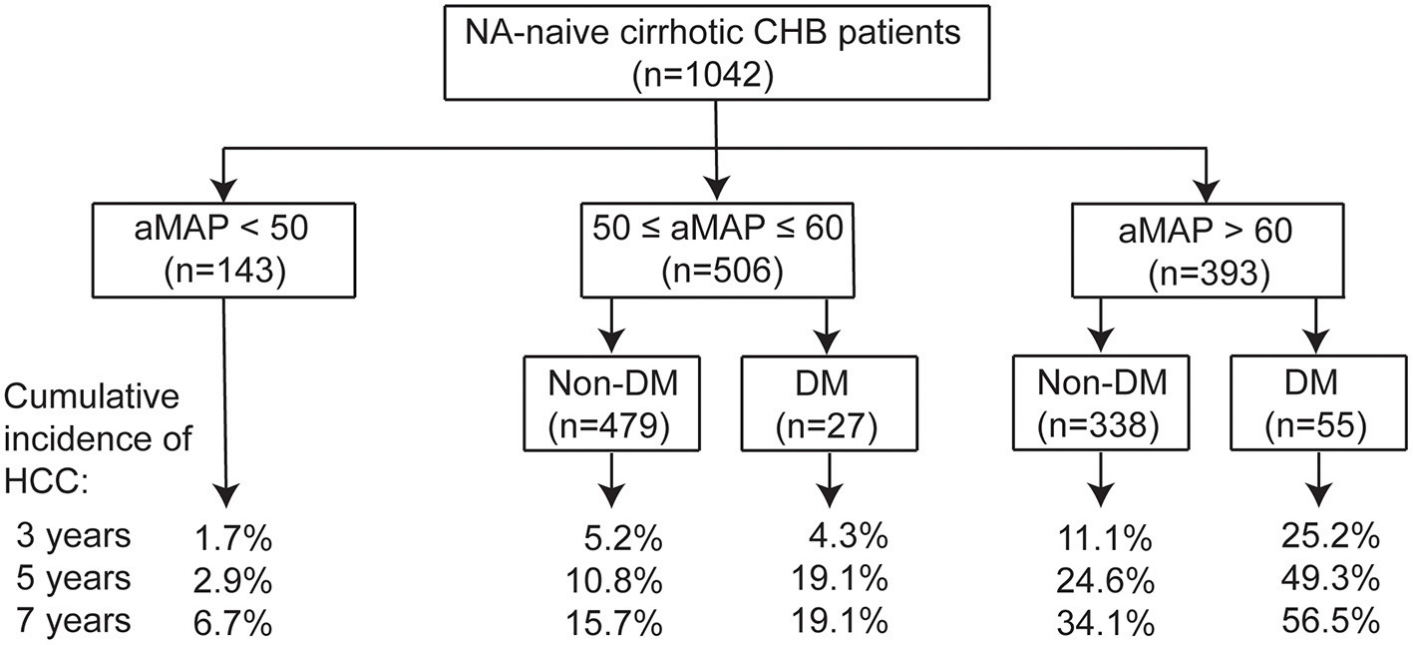
Proposed algorithm for prediction of HCC risk by combination of aMAP and DM in NA-naïve cirrhotic CHB patients.

Because DM was an independent predictor of HCC in our cirrhotic cohort, we investigated whether the combination of aMAP and DM could stratify the patients into subgroups of different HCC risks. As shown in Figure 3, patients in the aMAP high-risk group with DM (*n* = 55) exhibited the highest risk of HCC, with a cumulative incidence of 25.2, 49.3, and 56.5% at 3, 5, and 7 years, respectively, while patients with aMAP >60 who did not have DM (*n* = 338) had an intermediate-high risk of developing HCC (*p* < 0.001). The HCC risk did not differ significantly between patients with (*n* = 27) and without DM (*n* = 479) in the aMAP intermediate-risk group (*p* = 0.5). The addition of DM status did not further stratify patients in the aMAP low-risk group. On the basis of these findings, we proposed a clinical algorithm for predicting HCC risk in patients with CHB cirrhosis who undergo NA therapy (Figure 4).

### Performance of aMAP Score During NA Therapy

As shown in Table 5, the values of the four risk scores calculated 1 year after the initiation of antiviral therapy showed significant changes compared to those calculated at baseline (all *p* < 0.05). The predictive performances with reassessment of both the aMAP (Harrell's c-index 0.746, 95% CI 0.723–0.769) and mPAGE-B (Harrell's c-index 0.744, 95% CI 0.721–0.766) scores outperformed significantly in comparison with those calculated at the enrollment. There were no significant differences in the CAMD or PAGE-B scores calculated at baseline or 1 year after therapy. As shown in Table 1, higher 1-year aMAP scores were observed in patients with HCC; however, the proportion of patients with ΔaMAP ≥ 0 was not significantly different between the patients with HCC and those without HCC. That was to say, cirrhotic patients with ΔaMAP <0 did not exhibit a lower HCC risk than those with ΔaMAP ≥ 0.

**Table 5:** The predictive performances of HCC risk scores after 1-year antiviral therapy.

**HCC risk scores**	**Values^a^**	**Harrell's c-index**	**95% CI**
aMAP	56.7 ± 7.25^****^	0.746	0.723–0.769
CAMD	14.2 ± 2.35^****^	0.720	0.697–0.742
PAGE	15.6 ± 4.25^*^	0.705	0.679–0.730
mPAGE	11.5 ± 3.06^****^	0.744	0.721–0.766
**Comparison(baseline vs. 1-year)**	**Difference between Harrell's c-indices of models**		**95% CI^b^**
aMAP	0.021		0.003–0.040
CAMD	0.001		−0.013–0.015^b^
PAGE-B	0.009		−0.014–0.034^b^
mPAGE-B	0.025		0.004–0.046

*HCC, hepatocellular carcinoma; CI, confidence interval*.

a *Comparison of values of HCC risk scores between baseline vs. 1-year*,

* *p < 0.05*,

**** *p < 0.0001*.

b *If 95% CI interval contains zero, there is no significant difference between two models*.

## Discussion

Although the effect of antiviral therapy on reducing the risk of HCC development has been well-established, the substantial residual risk of HCC during long-term NA therapy may still exists, particularly in patients with cirrhosis (20). It is critical to determine a more accurate HCC prediction model optimized for disease management in at-risk patients with CHB-related cirrhosis. In this independent cohort study, we validated that the aMAP score has a superior or comparable predictive performance (Harrell's c-index: 0.724; 95% CI: 0.701–0.747) in predicting HCC development among patients with cirrhosis undergoing long-term ETV or TDF therapy. The large sample size and mid-long-term follow-up in this study enhance the statistical reliability of the results. Moreover, a sufficient number of patients developed HCC during follow-up, affording adequate statistical power. The relative homogeneity of our study population, well-defined population of CHB with compensated cirrhosis receiving ETV or TDF therapy, reduced potential confounding factors.

Older age, male sex, and lower platelet count are well-known risk factors associated with HCC development (13, 15, 16). In addition, higher serum total bilirubin levels are an independent risk factor for HCC development in this study, which is consistent with previous studies (8, 16). In this study, lower serum albumin levels were not found to be an independent risk factor for HCC development. This is most likely due to the fact that patients with decompensated or advanced cirrhosis (Child-Pugh class B or C) were excluded from our study.

Liver cirrhosis has been reported to be a risk factor for HCC development in patients with CHB with or without NA therapy (7, 14). In the present study, all patients had cirrhosis at enrollment based on liver biopsies or clinical, radiographic, and/or laboratory data. A clinical diagnosis of cirrhosis is more likely in real-world practice. Furthermore, the fact that cirrhosis is not included in the aMAP score is likely advantageous as long-term NA therapy can lead to the regression of histological cirrhosis in patients with CHB (21). The significantly improved predictive performance of the aMAP score after 1 year of NA therapy compared to its calculated at baseline may be explained by the specific variables included in the aMAP score. In other words, NA therapy as a risk modifier of HCC may have a salutary effect on the aMAP score as well. However, cirrhotic patients with a decline in aMAP from baseline to after 1 year of treatment did not exhibit a lower HCC risk than did those with an increased aMAP in our study. Consequently, a single short-term on-treatment change of aMAP was insufficient to determine the risk of HCC. As for the issue of whether the HCC risk was only apparent when aMAP scores moved between categories, future studies that combine aMAP and ΔaMAP will be better positioned to address it. Notably, our study validated that the aMAP score could predict HCC risk not only in the first 5 years of therapy, but also after 5 years of therapy. Since the aMAP score is easily computed using data from routine laboratory tests, it can be monitored regularly in patients undergoing NA therapy and meets the predictive needs of clinical practice.

The predictive performance of the aMAP score was comparable to that of the CAMD and mPAGE-B scores models and better than that of the PAGE-B score. The aMAP score may be useful for identifying a subgroup of patients with cirrhosis with a low risk of the development of HCC (aMAP <50 indicates a 2.9% risk of HCC within 5 years). Typically, surveillance strategies to detect early-stage HCC are cost-effective when the annual risk exceeds 0.2% in patients without cirrhosis and 1.5% in patients with cirrhosis. Therefore, the low-risk subgroup (which accounts for 14% of the study population) with an HCC incidence of <1.5%/year indicates the need to recalculate the cost-effectiveness of the current HCC surveillance practices. Patients with an aMAP score <50 achieved an NPV of 96.6% for the 5-year development of HCC, which is similar to the reported NPV of patients with cirrhosis when the PAGE-B related scores are used (22). When a more precise definition of low HCC risk is created by combining the aMAP and mPAGE-B scores, 11.6% of patients with cirrhosis undergoing NA therapy were classified as low risk with NPVs of 100 and 98.2% to exclude HCC at 3 and 5 years, respectively. Therefore, life-long HCC surveillance is recommended for patients with cirrhosis. Future research efforts will focus on exploring additional biomarkers to further identify patients with zero risk of HCC development for whom HCC surveillance may no longer be needed or may be able to be performed at longer intervals.

HCC risk scoring systems are also useful to identify patients with a high risk of HCC development and a potential need for intensified HCC surveillance. HCC surveillance is currently based on biannual abdominal ultrasounds for all patients with cirrhosis, regardless of the patient's actual HCC risk. This approach often results in an over- or underestimation of HCC risk for each patient (23). Additionally, the diagnostic accuracy of ultrasound is suboptimal in patients with cirrhosis, particularly for the detection of early-stage HCC. Dynamic CT or MRI offer higher sensitivity and specificity in detecting HCC, but have several limitations for general use in HCC surveillance (24). Therefore, patients with cirrhosis who are classified in the high-risk group may be candidates for specific HCC surveillance with more sensitive imaging modalities such as MRI (25). HCC risk scores offer clinically useful information by identifying subgroups of patients with CHB with an annual HCC risk exceeding 3–5% (17). In the present study, 37.3% of patients with cirrhosis undergoing NA therapy were classified in the high-risk group and among these patients 27.9% developed HCC at year 5. Like existing HCC risk scores, the PPV of the aMAP score at a cut-off point of 60 was not optimal. Moreover, the use of the “AND” and “OR” approaches with aMAP and other HCC scoring systems was not able to reach optimal PPV.

DM or prediabetes, as an important component of metabolic syndrome, has been found to be associated with an increased incidence of HCC (26, 27). CHB Patients with newly diagnosis of DM have an increased risk of cirrhosis and hepatic decompensation over time (28). DM is a risk factor for HCC development in patients with CHB and cirrhosis who undergo NA therapy (29). The association between DM and HCC risk has been reported to be independent of cirrhosis, although most patients with HCC presented with cirrhosis (30). In fact, it seems that DM is a bigger independent risk factor for HCC than any of the other variables examined in this study. Interestingly, further analysis of combination of DM and aMAP found that DM could help to stratify the aMAP high-risk group, but not the low- or intermediate-risk groups. The statistically inconsistent effect across aMAP risk groups might actually be attributed to confounding by other underlying variables (e.g., metabolic liver disease), that may be hard to determine within the limits of our dataset. It should also be noted that there is a large disparity in the proportions of diabetic patients among different aMAP risk groups in our study, 2.1, 5.3, and 14.0% in low-, intermediate-, and high-risk groups, respectively.

The biological mechanism for how DM influences HCC development is not well-understood, but several possibilities have been hypothesized. Insulin resistance and hyperinsulinemia, hallmarks of type 2 diabetes, are believed to play important roles in hepatocarcinogenesis. DM is also an important risk factor for the development of steatohepatitis, which may be associated with an increased risk of HCC. As DM is easy to assess in clinical practice and it significantly improves the predictive value of the aMAP score, we recommend routine screening for DM in patients with CHB with or without cirrhosis before the initiation of NA therapy. Metformin, which is administered to improve insulin sensitivity, has been reported to be associated with decreased HCC risk (31). Therefore, the proper management of DM and cautious selection of therapy for DM in patients with CHB, especially for those with a high risk of cirrhosis, are recommended.

This study is not without limitations. First, the present study was conducted solely based on a retrospective, single-center cohort in a large tertiary referral university hospital, and its results are potentially subject to selection bias. Second, although we validated the predictive performance of the initial aMAP score and the 1-year aMAP score, the predictive role of the combination of aMAP and on-treatment changes in aMAP (ΔaMAP) during NA therapy and their optimal time point requires further investigation, as in the case of the fibrosis index based on four factors (FIB-4) (32). Third, given that metabolic risk factors, including diabetes, obesity, high blood pressure, and hypercholesterolemia, are associated with an increased HCC risk in patients with CHB (33), other metabolic risk factors should be incorporated in future studies as such models will have higher discriminatory power. In addition, future research to determine how the control of glycemia, blood pressure, and lipids and/or the types of antidiabetics, antihypertensive, and lipid-lowering medications may affect the risk of HCC is required. Finally, the severity of liver fibrosis/cirrhosis and the longitudinal changes during NA therapy were not evaluated by transient elastography (TE) in this study as this technology was not available for the majority of the study period in our hospital. In addition, it is difficult to perform paired TE examinations in a large cohort of patients with CHB before and after long-term antiviral treatment in a real-life setting. The implementation of TE will likely improve the predictive performance of the aMAP score, similar to its effect on the LSM-HCC (10) and mREACH-B (12) scores.

In conclusion, the aMAP score achieved an acceptable and comparable HCC predictive performance in at-risk patients with compensated cirrhosis undergoing ETV or TDF therapy, not only calculated at baseline, but after 1 year of antiviral therapy as well. The addition of DM as a constituent to the aMAP score contributed to a marginal improvement in the prediction of extremely high HCC risk. These patients are suggested to undergo more intensive and effective HCC surveillance program for early diagnosis and to improve prognosis. In addition, these patients are optimal candidates for HCC chemopreventive agents, including metformin (31), interferon (34), and other therapies that may be developed.

## Data Availability Statement

The raw data supporting the conclusions of this article will be made available by the authors, without undue reservation.

## Ethics Statement

The studies involving human participants were reviewed and approved by the Research Ethics Committee of Ruijin Hospital. Written informed consent for participation was not required for this study in accordance with the national legislation and the institutional requirements.

## Author Contributions

QX and HG conceived and designed the study. HG, YH, GZ, LC, WC, and HW collected the clinical data. HG, YH, and QG conducted the analysis and prepared the manuscript. QG and QX critically revised the manuscript. All authors approved the final version of the manuscript.

## Conflict of Interest

The authors declare that the research was conducted in the absence of any commercial or financial relationships that could be construed as a potential conflict of interest.

## Publisher's Note

All claims expressed in this article are solely those of the authors and do not necessarily represent those of their affiliated organizations, or those of the publisher, the editors and the reviewers. Any product that may be evaluated in this article, or claim that may be made by its manufacturer, is not guaranteed or endorsed by the publisher.
