# Supplementary material for: External Validation of aMAP Hepatocellular Carcinoma Risk Score in Patients With Chronic Hepatitis B-Related Cirrhosis Receiving ETV or TDF Therapy
Source: Front Med (Lausanne). 2021 Aug 4;8:677920. doi: 10.3389/fmed.2021.677920 (PMC8371628; doi:10.3389/fmed.2021.677920)
Supplement: Supplementary file 1 [file Table_1.DOCX]

Supplementary table 1. Variables and calculation of the four HCC risk prediction models

| Risk score | Calculation | Cut-off value |
| --- | --- | --- |
| CAMD | Cirrhosis (no cirrhosis =0; cirrhosis at age <40y =10; cirrhosis at age ≥40y =6) + age (<40 years =0; 40-49 years =5; 50-59 years =8; ≥60 years =10) + gender (male =2; female =0) + Diabetes (no =0; yes =1) | Low-risk: <8  Medium-risk: 8-13  High-risk: >13 |
| PAGE-B | Gender (male =6; female =0) + age (16-29 years =0; 30-39 years =2; 40-49 years =4; 50-59 years =6; 60-69 years =8; ≥70 years =10) + platelet (≥200,000/mm^3^=0; 100,000-199,999/mm^3^ =6; ≤100,000/mm^3^ =9) | Low-risk: <10  Medium-risk: 10-17  High-risk: >17 |
| mPAGE-B | Gender (male =2; female =0) + age (<30 years =0; 30-39 years =3; 40-49 years =5; 50-59 years =7; 60-69 years =9; ≥70 years =11) + platelet (≥250 =0; 200-250 =2; 150-200 =3; 100-150 =4; <100 =5) + albumin (≥4.0 =0; 3.5-4.0 =1; 3.0-3.5 =2; <3 =3)  albumin is in g/dL and platelets in 10^3^/mm^3^. | Low-risk: <9  Medium-risk: 9-12  High-risk: >12 |
| aMAP | ((0.06 × age + 0.89 × sex (Male: 1, Female: 0) + 0.48 × ((log_10_bilirubin × 0.66) + (albumin × -0.085)) -0.01 × platelets) +7.4) /14.77 × 100  age is in year, bilirubin in μmol/L, albumin in g/L and platelets in 10^3^/mm^3^. | Low-risk: <50  Medium-risk: 50-60  High-risk: >60 |
